# On-Farm Claw Scoring in Sows Using a Novel Mobile Device

**DOI:** 10.3390/s19061473

**Published:** 2019-03-26

**Authors:** Miriam M. J. van Riet, Jürgen Vangeyte, Geert P. J. Janssens, Bart Ampe, Elena Nalon, Emilie-Julie Bos, Liesbet Pluym, Frank A. M. Tuyttens, Dominiek Maes, Sam Millet

**Affiliations:** 1 Fisheries and Food (ILVO), Animal Sciences Unit, Flanders Research Institute for Agriculture, Scheldeweg 68, 9090 Melle, Belgium; miriam.vanriet@hotmail.com (M.M.J.v.R.); bart.ampe@ilvo.vlaanderen.be (B.A.); elena.nalon@gmail.com (E.N.); emiliejuliebos@gmail.com (E.-J.B.); frank.tuyttens@ilvo.vlaanderen.be (F.A.M.T.); 2 Faculty of Veterinary Medicine, Department of Nutrition, Genetics and Ethology, Ghent University, Heidestraat 19, 9820 Merelbeke, Belgium; geert.janssens@ugent.be; 3 Fisheries and Food (ILVO), Technology and Food Science Unit, Flanders Research Institute for Agriculture, Burgemeester Van Gansberghelaan 115, 9820 Merelbeke, Belgium; Jurgen.Vangeyte@ilvo.vlaanderen.be (J.V.); liesbet.pluym@vlam.be (L.P.); 4 Faculty of Veterinary Medicine, Obstetrics, Reproduction and Herd Health, Ghent University, Salisburylaan 133, 9820 Merelbeke, Belgium; Dominiek.Maes@ugent.be

**Keywords:** sows, claw lesions, claw scoring, farm, Mobile Claw Scoring Device

## Abstract

Claw lesions and lameness in sows are important problems in the industry as they impair sow welfare and result in economic losses. Available scoring techniques to detect claw lesions are all limited in terms of collecting data during all reproductive phases and recording all claws. The Mobile Claw Scoring Device (MCSD) was designed to address these limitations. After considering different practical situations and a design phase, two prototypes were constructed and tested. Improvements were incorporated into a final aluminium apparatus, consisting of two cameras with light-emitting diode (LED) lights mounted in a two-segment aluminium box and covered with laminated tempered glass plates. The operating system slides underneath the claws and takes video images. This final prototype was optimised and validated in an experiment with 20 hybrid sows, comparing scores for soiled claws using the MCSD against scores for clean claws using the Feet First^©^ sow chute (as gold standard). Fifty percent of the scores differed between both scoring tools, with mainly medial claw digits deviating, but this seemed biologically irrelevant. The MCSD seems to be an appropriate alternative for on-farm claw scoring and is distinguishable from other claw scoring techniques; however, it needs further optimisation to improve the similarity between the two techniques.

## 1. Introduction

Claw lesions and lameness are a main reason for culling sows before they reach their full breeding potential and results in both impaired welfare and considerable economic losses [1,2,3,4,5,6]. Furthermore, reproductive failure and culling negatively affects performance and represents additional costs due to extra labour, medication required to treat the sow, and pre-culling (e.g., costs related to culling a sow before reaching her full breeding potential and the new gilt to replace her) [4]. Almost every sow has one or more claw lesions, depending on type and severity [7,8,9]. As expected, the prevalence of sows with one or more claw lesions is high, varying between 50% and 100% [9,10,11,12,13]. The prevalence may have increased since group housing for gestating sows became mandatory in the European Union (EU) in January 2013. This legislation has had a positive impact on welfare and behaviour, but the increased (hierarchical) interactions among sows can lead to falls and slipping, which can damage the legs and claws [7,8,9,10,14]. The prevalence of lameness is lower, estimated at around 10% in Western Europe [15,16,17]. 

The most frequently observed claw lesions in sows, varying in severity, are heel horn erosions, defects in the heel horn/sole junction, white line defects, horizontal and vertical wall cracks, claw and dewclaw overgrowth, ulcers, and skin lesions [18,19,20]. Claw lesions have a multifactorial origin, including behaviour, locomotion disorders (5%–20% of cases [1,2,5]), floor type [9,10,21,22,23,24,25,26], nutrition [27,28,29,30,31,32,33,34], and different management systems as predisposing factors. These predisposing factors affect claw quality internally or externally by inducing inflammation, trauma, and/or mechanically inferior horn, resulting in disrupted claw development and integrity that eventually leads to lesions [3]. However, it remains unclear to which extent these factors or a combination of factors cause claw lesions. For this reason, it is important that claw lesions and their prevalence are recorded, which in turn will allow for the analysis of (other) potential causative factors and preventive strategies.

**Figure 1:**
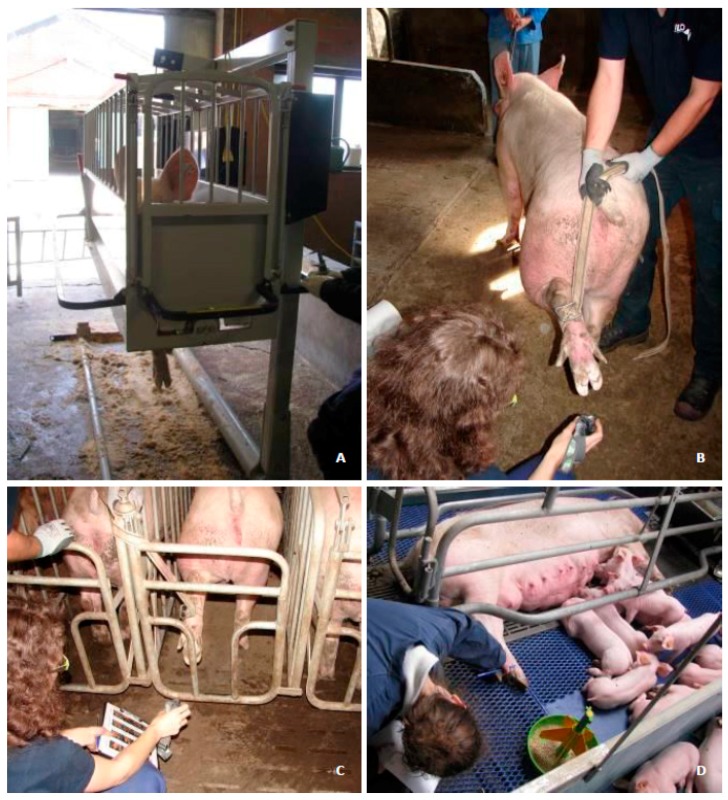
Different techniques for visualising claw lesions in pigs. (**A**) Claw scoring using the sow chute (with permission of © ZINPRO corporation, Eden Prairie, MN, USA, www.zinpro.com). (**B**) Scoring the hind claws with the sow standing outside the crate, requiring snaring and lifting the hind leg(s). (**C**) Scoring the hind claws with the sow standing in the crate, thereby lifting the hind leg(s). (**D**) Claw scoring in the farrowing crates when the sow is lying down.

Claws can be scored for claw lesions using the same scoring methods, but different techniques, including: (1) Claw scoring in the farrowing crates when the sow is lying down; (2) scoring the hind claws with the sow standing in the crate by lifting the hind leg(s); (3) scoring the hind claws with the sow standing outside the crate, which requires snaring and lifting the hind legs; and (4) via the Feet First^©^ chute (“sow chute”; Zinpro Corporation) (Figure 1). These techniques differ in their practical implications. They vary in terms of the labour required, stress on the sows, degree of accuracy in viewing the claws, disinfection, and use during all reproductive phases. Important limitations for all these techniques are their usefulness to collect data from both the front and hind claws on-farm, since the techniques are either used in the farrowing crate or require the sows to be restrained, possibly impairing their welfare. 

These limitations call for the development of a new technique for on-farm claw scoring that is designed to reduce sow stress and enable sows to stay in their familiar surroundings. Furthermore, this new technology should be easy to handle and transport and the claw scoring should only take a few minutes. Another important specification should be that both front and hind claws can be scored. After the new technique is designed, it needs to be validated. The claw lesion scores obtained from the new scoring technique need to be compared against a gold standard with the hypothesis that the claw lesion scores for this new technique do not differ from the scores noted from the gold standard. For this experiment, we considered the manual inspection of the claws from a sow hoisted up in the Feet First© sow chute as the gold standard for assessing claws.

## 2. Materials and Methods 

To develop an effective and differentiated technique for on-farm claw scoring, the following specifications were required for the Mobile Claw Scoring Device (MCSD):

- The sow remains in familiar surroundings without snaring, presumably reducing stress levels.
- Easy to handle, disinfect, and transport.
- Fast claw scoring without needing to clean the claws.
- Claw scoring in either real time or afterwards based on recorded images.
- Used during multiple phases of the reproductive cycle.
- On-farm scoring of both front and hind claws.

**Figure 2:**
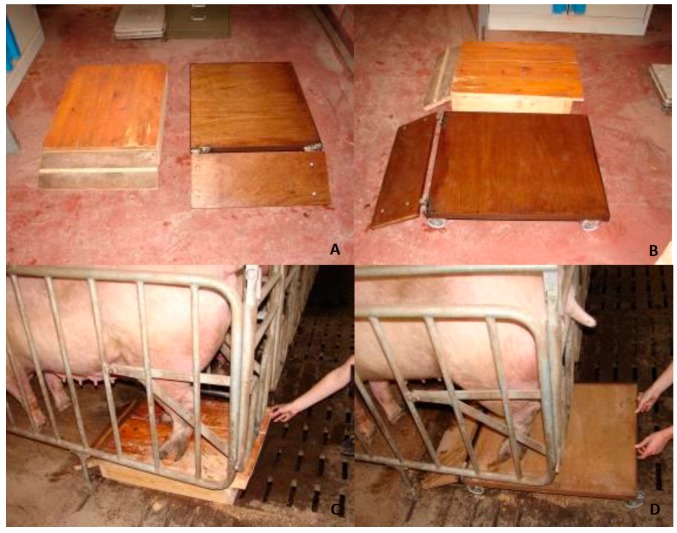
First and second wooden prototypes of the Mobile Claw Scoring Device (MCSD). (**A**,**B**) = top and side views of the prototypes. The left MCSD is the first and the right MSCD the second prototype. (**C**) The first wooden prototype tested in a gestation crate. (**D**) The second wooden prototype tested in a gestation crate.

First, the conditions and feasibility of the MCSD were assessed by determining the minimum and maximum applicable specifications for its use in different housing systems. First common housing systems in the pig industry were evaluated and crate dimensions were measured. Based on these crate measurements, the average dimensions were 700 mm × 600 mm (length (l) × width (w)) with a maximal height of 150 mm. To fit in most conventional housing systems, the MCSD should not exceed these dimensions. To test usability at various locations on the farm and the willingness of the sows to cooperate, two wooden prototypes were empirically designed and used (Figure 2). These prototypes consisted of a wooden frame with or without wheels and with or without a hinged oblique plate. Based on tests with the wooden prototypes, moving a sow towards the MCSD was not practical. If the sow was moved towards the front of the crate simultaneously with placing the MCSD into the crate and encouraging the sow to move backwards, she was more willing to step onto the MCSD. This procedure was tested in individual (gestation) crates as well as in farrowing crates at Flanders Research Institute for Agriculture, Fisheries and Food (ILVO’s) experimental farm. 

**Figure 3:**
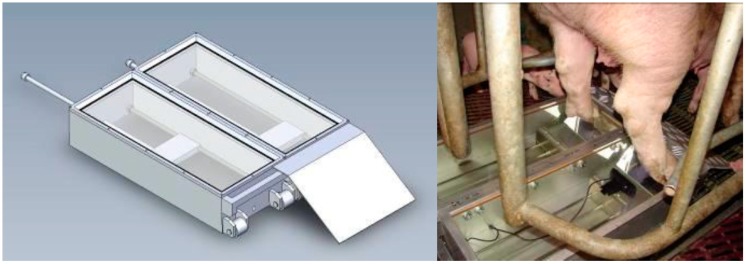
The third prototype of the Mobile Claw Scoring Device (MCSD) presented via the CAD-software (**left**) and at the first trial in the farrowing crates on the ILVO experimental farm (**right**).

**Figure 4:**
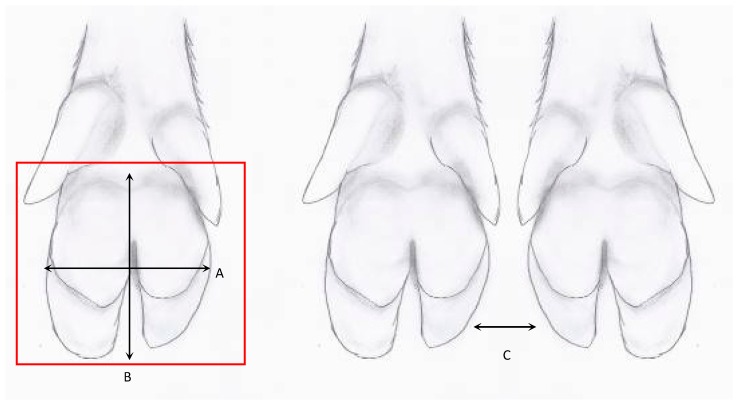
Claw anatomy of pigs to calculate the surface area that must be covered by the cameras for a complete view of the claw. The red coloured square represents the area of the claw which is often in contact with the underlying surface when standing and moving. A = the claw width varying between 65 and 80 mm, and B = claw length varying between 95 and 130 mm. Based on these claw measurements, the calculated surface area is 130 mm × 80 mm (i.e., 10,400 mm^2^). C = the maximum distance between the cameras, which was determined by measuring the distance between the left and right claw, which was 230 mm, range was 70 to 230 mm.

An aluminium MCSD prototype (third prototype, Figure 3) was then developed to investigate the stability of the apparatus, cleaning and disinfection, weight load of the glass plates, and required specifications of the cameras. The dimensions were set at 2 times 600 mm × 250 mm × 130 mm (l × w × h). To test the cleaning and disinfection procedure, glass plates with the same features as used in the MCSD were divided into areas on which several disinfectants and concentrations per disinfectant were added. The disinfectants tested were similar to products used on farms, eliminating the need to purchase extra products. For the cameras, the surface area that the cameras had to cover for a complete view of the claw was calculated based on the width and length of claws. The maximum surface area to cover was 130 mm × 80 mm (l × w) per claw, with a maximum distance between cameras of 230 mm (i.e., maximum distance between the left and right claw) (Figure 4). The cameras needed to reach this range by sliding on a rod within the maximal height of the MCSD of 130 mm. Other required specifications for the cameras were the capacity to record videos and take photographs through tempered laminated safety glass, and the camera placement at the bottom or at a maximum angle of 22° while still covering the required range. The cameras needed to have an appropriate size and weight, preferably with adjustments for colour, form, and reflection, be equipped with a non-reflective filter, and be robust enough to cope with vibrations when moving the MCSD over the (crate) ground or during transport. Several cameras were tested, including a Logitech webcam and digital cameras with different lens angles with or without the use of a mirror. Most of them could not record the complete surface area within the maximum height of 130 mm without extra light sources to illuminate the claws. The use of light sources improved the images, but reflections were visible in some cases. A light beam that runs along with the cameras was the best option. The use of a mirror did not add extra visibility and the mirror needed a certain distance from the cameras to project the view of the claw. This required distance between the mirror and cameras limited the covered range of the cameras even further relative to the length of the MCSD. To ensure that the cameras were able to record the claw, irrespective of the position of the claw on the glass plate, the cameras needed to move along the full length of the MCSD. Introducing an operating system with a rod enabled each camera to slide to the position of the claws on the glass plates, even when the sow was standing on a corner of the MCSD. The use of a mirror still limited the range and was therefore not implemented in the definitive design. After these (technical) considerations, the third prototype was tested at the ILVO’s experimental farm. The procedure remained the same: The sow was encouraged to move backwards to stand on the glass plates with her hind legs. The cameras were then positioned under the claws and the claw features were recorded. The same procedure can be repeated to record the front claws. Between sows, the MCSD was cleaned with water and dried using a microfibre towel. Before every farm visit, the MCSD is expected to be disinfected and dried with a microfiber towel. After testing the third prototype, further adjustments were implemented, including a detachable aluminium tear plate (as ramp) with transverse strips, so that the sow has a better grip when walking backwards onto the MCSD. The handle at the side of the tear plate was then also covered to prevent the sow from stepping on the handle. With the handle located at the side of the tear plate and at the back of the MCSD, the MCSD could be hoisted up. 

**Figure 5:**
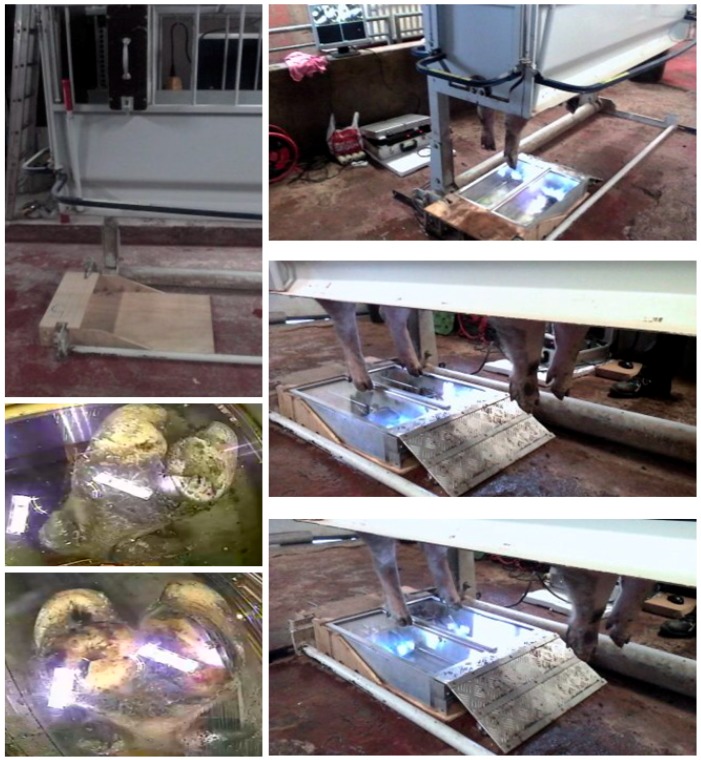
Experimental design for validating the Mobile Claw Scoring Device (MCSD). A wooden cover frame fitted over the core of the sow chute in which the MCSD was inserted to stabilise and protect against damage to the MCSD when sows entered the sow chute (with permission of © ZINPRO corporation, Eden Prairie, MN, USA, www.zinpro.com). When the sow stood on the MCSD, each camera was manually slid on its own rod to adjust to the position of the claws on the glass plates. Two snapshots were taken from the video recordings to visualise the view of the claws from the cameras.

**Figure 6:**
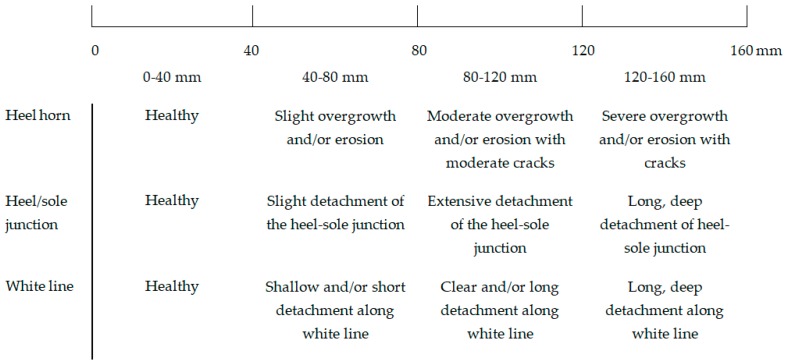
Tagged visual analogue scale (tVAS) for claw lesion scoring in sows, adapted and modified from the scorings methods of Wageningen University and FeetFirst (Zinpro Corp., Eden Prairie, MN) as described in [9,10,31]. To score the claw area for claw lesions, a vertical bar was drawn on the line and the distance from 0 mm determined, reflecting the severity of a claw lesion.

All gathered findings were integrated into the definitive design using Computer Aided Design (CAD) software and constructed using the third prototype as the basis. The definitive design was validated relative to the Feet First^©^ chute (“sow chute”; Zinpro Corporation, Eden Prairie, MN, USA (chosen as the gold standard for this experiment) because in the sow chute, the front and hind claws are fully visible and can be cleaned properly. This experiment was conducted according to the institutional and national guidelines for the care and use of animals. ILVO’s Ethics Committee for animal experiments (approval no. 2014/236, 5 December 2014) approved all experimental procedures. For the experimental design, the MCSD dimensions made it possible to place the MCSD in the sow chute using a wooden cover frame to prevent damage to the MCSD when sows entered the sow chute (Figure 5). By positioning the MCSD in the sow chute, the chute tray was located 130 mm higher from the ground than normal, but the sows showed no reluctance to step over this height and no problems were encountered. Twenty mid-gestating hybrid sows (RA-SE Genetics, day 28–60 of gestation) from one group with an average parity of 1.45 ± 0.6 (±SD) were used in this study. The sows were group-housed and had ad libitum access to water. The sows were fed 2.6 kg/d of a commercial gestation diet using an electronic feeding system (Nedap, Groenlo, The Netherlands). Each sow was separated from the group and placed in the sow chute for 8 ± 1.4 min (±SD). Video recordings of the left and right hind claws for the MCSD were taken immediately, and then the sow was hoisted up using the sow chute. The hind claws were cleaned with water and a brush, then paper towels were used to dry the claws. As needed, a hoof knife was used to remove sticking manure. Both hind claws were scored visually by three trained observers, representing the claw lesion scores of cleaned claws in the sow chute. In total, 2 observations per sow per observer were conducted (soiled claws × MCSD and clean claws × sow chute). Scoring soiled claws with the MCSD were tested against scoring cleaned claws in the sow chute as the gold standard, because the MCSD is especially designed for on-farm use where claws are often soiled. To validate if the soiled claw scores obtained from the MCSD were correct, scores needed to be compared against the scores of clean claws obtained from the sow chute where claws were clearly visible and the smallest cracks and separations were detectable. The recordings of the MCSD were scored manually for claw lesions in two sessions; recordings of the claws from the first 10 sows were assessed 35 days after scoring the hind claws in the sow chute and collection of the video recordings. The recordings of the 10 remaining sows were assessed 83 days after collection. This time-lapse was intentional; it was meant to reduce the chance that the observers would remember specific claw lesions. The order of sows for claw lesion scoring was the same for the sow chute and for the video recordings of the MCSD. For claw lesion scoring, a scoring method adapted and modified from the FeetFirst scoring guide (Zinpro Corp, Eden Prairie, MN, USA) and the “Zeugenklauwen Check” (Wageningen University) was used [9,10,31]. This method represents a tagged visual analogue scale (tVAS) of 160 mm with descriptors on 40, 80, and 120 mm indicating the severity of a claw lesion type, instead of an ordinal scoring scale presented in the literature. To score the claw for a claw lesion, a vertical bar was drawn on the line and the distance from 0 mm was determined, thus reflecting the severity of a claw lesion (0 mm is completely healthy and 160 mm most deteriorating) [9,10,31]. In this way, the lateral and medial claw digits from both hind claws were scored for the occurrence and severity of three types of claw lesions, namely heel horn erosion, separations along the heel-sole junction, and separations along the white line (Figure 6). Three trained observers scored all claws and had experience with claw lesion scoring for over 4 years. The inter- and intra-observer reliabilities were tested in a previous experiment and varied between 0.48 and 0.71 and between 0.55 and 0.75, respectively, for the tested claw lesion types in this experiment (under review). In total, 60 observations (20 sows × 3 observers) per outcome variable (score for lesion type on lateral or medial claw digit on left or right hind claw) per scoring technique were recorded and further analysed. Additionally, the average of all lateral claw lesion scores (within sow and observer), the average of all medial claw lesion scores, and the overall average of all claw lesions scores were calculated. All values were added in the Microsoft Excel dataset and further analysed to test the hypothesis that the tVAS scores of the MCSD with soiled claws equals the tVAS score of the sow chute with clean claws. The individual tVAS claw lesion scores and average scores were analysed using a linear mixed model. The technique of scoring (sow chute × clean claws or MCSD × soiled claws) and the observer were included as fixed effects, while the sow was included as a random effect to correct for repeated measurements. Non-significant main effects were not excluded from the final models. In the case of post hoc pairwise testing, *p*-values were corrected with the Tukey-Kramer adjustment for multiple comparisons. The analysed data were considered to be sufficiently normally distributed based on the graphical evaluation (histogram and QQ-plot) of the residuals. All analyses were performed using SAS 9.4 (SAS Institute Inc., Cary, NC, USA).

## 3. Results

### 3.1. Definitive MCSD Characteristics

**Figure 7:**
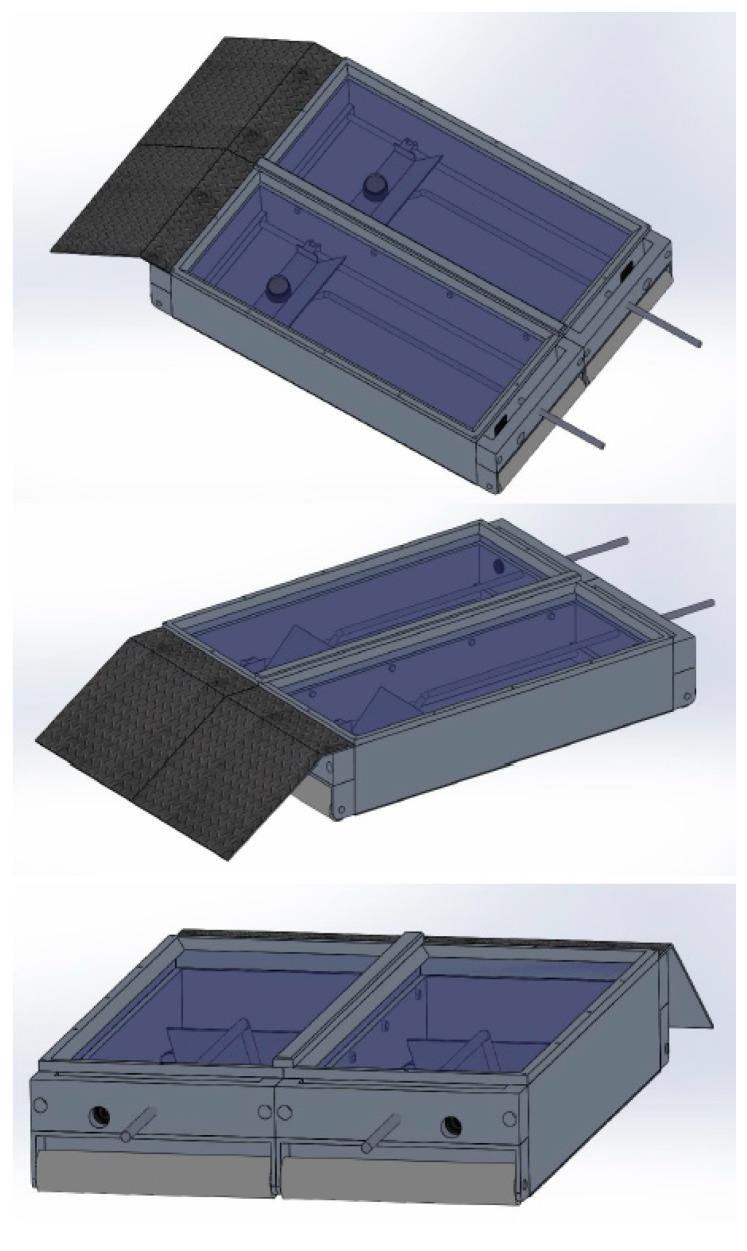
Current MCSD after adaptions of the three previous prototypes.

The specifications of the definitive MCSD are summarised in Table 1 and Figure 7. The MCSD consists of two aluminium detachable boxes to ensure easy handling and transport. During use, the boxes are fixed to each other using stainless steel pins with a locking slot and slide-in frame. The total weight of the MCSD is 33.3 kg exclusive of the cameras and tear plate. In the front of the MCSD, an oblique aluminium tear plate with transverse strips is added to facilitate the sow stepping onto the MCSD. A roller is attached underneath the tear plate, at the front and at the back of the MCSD, for easy and smooth placement of the MCSD in the crates. The glass plates are placed in the aluminium box using para rubber and are held in place by an aluminium frame attached to the aluminium box by 12 RVS A2 hexagonal recessed head screws (3.0 Ø × 20 mm). The movable operating system with one camera (wide-angle mini dome camera, Model IQ667, IQLE, Harderwijk, The Netherlands) in each box is installed underneath the glass plate. Each camera can manually slide on its own rod when adjusting the position of the cameras to the position of the claws on the glass plates. Each camera is connected to a water-resistant transceiver in the aluminium wall and the continuous signal via a high-definition multimedia interface (HDMI) wire is transduced to the digital video recorder (DVR, Oosterberg B.V., Ede, The Netherlands) with a resolution of 2592 × 1526 and a frame rate of 30 frames per second. The continuous video recordings were monitored by the DVR and saved as .dav files. A PC screen was connected to the DVR to determine whether the claw was in fact visible on the video recording. 

### 3.2. Validation of MCSD

Differences between the claw lesions scores of the cleaned claws obtained from the sow chute and the soiled claws obtained from the MCSD were found for 6 out of 12 scores (Table 2); 4 for medial and 2 for lateral claw digits, primarily for scores of the separation along the white line (Table 2). The heel horn erosion scores of the medial claw digits of both the left and right hind claws were higher (meaning more erosion) for the MCSD. Likewise, the MCSD showed higher tVAS values than the sow chute for the (1) separation along the heel-sole junction of the medial digit of the left hind claw and (2) separation along the white line of the medial digit of the left hind claw. The tVAS scores for separation along the white line of the lateral claw digit of the left and right hind claws were higher for the sow chute than for the MCSD. 

The average claw lesion scores for the lateral claw digit and for the overall mean claw lesion score did not differ between the sow chute and the MCSD (Table 2). The average claw lesion score for the medial claw digit differed. This estimated difference was 8.23 mm on a 160 mm tVAS scoring scale, showing higher tVAS values for the MCSD.

Differences between observers were found for all claw lesion types on the lateral and medial claw digit of the left and right hind claw, except for separation along the white line of the medial claw digit of the left hind claw. Furthermore, differences between observers were also found for the average claw lesion scores for the lateral claw digit, medial claw digit, and for the overall mean.

## 4. Discussion and Conclusion

Fifty percent of the claw lesion scores differed between the MCSD and sow chute. In addition, the average claw lesion score for the medial claw digit differed, but did not differ for the lateral claw digit and overall mean. Although the differences reached significance, the question arises how relevant these differences are. Only two scores had a difference above 10 mm (11.85 and 14.41 mm). On the 160 mm tVAS scale, this means that the difference of the above scores is 7.4% and 9.0%, respectively. Expressed in terms of the adjusted 98 mm range, based on the minimum of 2 mm and maximum of 100 mm scores given during the experiment, the above differences were 12.1% and 14.7%. To our knowledge, 10 mm is presumably the lowest detectable distance on the tVAS to discriminate between scores, but 20 mm is probably more relevant as it is half the range between each claw lesion severity category of 40 mm. Furthermore, differences were found between the three observers, especially for lesion scores that differed between the two techniques. Therefore, the differences between scores seem not to be biologically relevant; however, the applicability of the MCSD for on-farm claw scoring still needs further optimisation. Caution is advised when assessing the medial claw digit and scoring for separations along the white line. Comparisons with the other scoring techniques indicated in Figure 1 were not tested in the present study. Furthermore, previous studies comparing claw scoring techniques are not available in pigs or other species. 

The MCSD has the required specifications to differentiate from the other techniques (Figure 1). Using the MCSD, the sow remains in her familiar surroundings and is not snared. This may induce less stress, although this was not tested in the present study. Also, in cows, the stress response differed between scoring techniques (standing position in a walk-in crush versus lateral recumbence on a tilt table) for claw trimming [35], in which a higher faecal cortisol metabolite concentration and more evasion movements were observed as indicators for stress using the walk-in crush. However, other claw trimming procedure steps may induce the stress response as well, including an interruption of the daily routine, the claw trimming itself with optical, acoustical, and tactile disturbances, and the handling of cows in the pre-trimming phase [36]. Although the claws of sows were not trimmed in the present study, the stress response is presumably lower using the MCSD when the sow remains in her crate compared to other techniques, were the sow is snared or placed in an unfamiliar chute with or without claw trimming. We recommend that future studies take this into account. 

Using the MCSD, claws can be scored either in real time or afterwards, which would save time during farm visits. Real time claw scoring using the MCSD was not evaluated during the validation experiment of the present study; however, it is expected that it requires more time on the farm. More relevant is the use of digital recordings (i.e., video recordings, which are assessed manually afterwards) versus direct manual scoring. Digital recordings were used in this validation experiment using the MCSD, while claws were directly manually scored using the sow chute. Differences, although probably biologically irrelevant, were found between the techniques; the impact of the type of scoring (digital recordings vs. direct manually) could not be distinguished in this experiment. In a study in cows testing five claw conformation measurements manually (directly from the hoof) vs. a digital image, results showed a large variation in the difference between the manual and digital claw conformation measurements. They concluded that these two methods could not be used interchangeably [37]. It appears that the type of images used can interfere with the outcome. However, this warrants further testing because it might be dependent on the variables tested and the observer. Laven et al. [37] tested the impact on claw conformation measurements, which is a more objective measurement compared with the more subjective visual claw lesion scoring used in the present study. Furthermore, in Laven et al.’s study [37], one observer determined the manual claw conformation measurements and another observer determined the digital measurements. Both observers used the same instructions, but there might have been some differences between observers. In this present study, all observers scored all sows; still, differences between observers have been observed, even though the three observers were trained and all had more than 4 years of experience in claw lesion scoring.

Differences in claw lesion scores for the medial claw digits were significant in four out of the total six instances, but it remains unclear why the medial claw digits had consistently worse scores using the MCSD. The digital recordings of the MCSD showed a full vision of both claw digits, with the lateral claw digits being further away from the centre of the camera than the medial claw digits. The sows preferred to stand close to the sides of the MCSD to have more grip, which might have changed their weight distribution. This in turn might have changed the print of the claw (darker and lighter spots), possibly changing the view. However, it is unknown to what extent this may have affected the scoring. 

When using the MCSD on-farm, some practical experiences need to be considered. The DVR was connected to the aluminium box of the MCSD using a cable, which sometimes obstructed the easy use of the MCSD. An electrical outlet must also be present in the stable. During the validation experiment, the observers often found it difficult to differentiate between a lesion and soiling of the claw. This applies for the MCSD and, to a lesser extent, for the sow chute. In the sow chute, claws can be manipulated, which might enable more accurate observations. Cleaning the claws with water, a brush, and a hoof knife might also improve the visibility of the claw, thus favouring the use of the sow chute. However, farmers do not have a sow chute and the claws are normally soiled during inspection. Another consideration is that the weight of the MCSD might hinder easy transportation. To address this issue, the two boxes can be dismounted after use and transported separately. The MCSD can be disinfected and, unlike the sow chute, can be used in various phases of the reproductive cycle. A farmer can also implement the MCSD in their management system by installing the MCSD in the electronic feeding station(s) during group housing. However, practical considerations and tests are needed to ensure clean glass plates. One limitation of the MSCD is that only the underside of the claws can be scored. Other claw lesion scores need to be scored manually on-farm, including skin lesions around the claw and dew claw, horizontal and vertical wall cracks, and the length of the claw and dewclaw. For these lesion scores, the cleanness of the claw is important to distinguish between a haemorrhage vs. soiling. When using software, some claw conformation measurements can be determined from the video recordings of the MCSD, including sole (base) length and claw width as described in van Riet et al. [30]. 

In conclusion, the MCSD seems to be an appropriate alternative when compared with the sow chute as a gold standard, but it still needs some optimisation. The MCSD does meet the specifications to be different from other claw scoring techniques. The MCSD can be used on-farm for claw lesion scoring in pigs, but recordings are only possible for claw lesions at the heel horn, at the heel horn-sole junction, and at the white line. The latter provides less reliable scores using the MCSD in comparison to the sow chute technique. Other claw lesion types can be scored while the sow is standing on the MCSD. Future research is warranted and should address the lighting (position, angle, quality) and camera quality of the MCSD to create better lighting conditions without reflection and to test the possibilities of infrared lighting. Furthermore, when the MCSD is used, the stress response of the sows must be determined and compared with other alternatives for claw lesion scoring. Last, claw scoring of the front claws needs to be validated.

## Figures and Tables

**Table 1:** Specifications of the Mobile Claw Scoring Device (MCSD).

**Aluminium box**	
Size (l × w × h)	Outside dimension per box: 600 mm × 250 mm × 130 mm
Weight (excl. glass plates)	22,500 gram
Material	20 mm thick aluminium (EN AW-6082, AlMgSi1, Demar-lux, Evergem, Belgium)
Oblique tear plate	290 mm Aluminium (EN AW-5754 H114)
Roll	White nylon roll under tear plate, at front and back of the MCSD
Connecting strip	Connecting the two aluminium boxes by using four stainless steel hex bolts in the side of the box and locked with a detachable aluminium connection strip
**Glass plates**	
Size (l × w)	Per box: 585 mm × 235 mm
Weight	10,760 gram
Material	8 mm tempered safety glass laminated with 4 plastic interlayers (EVM Glass Trading Company, Menen, Belgium) Antiglare Thickness: 17.52 mm Smoothed borders
Tolerance	2 mm
Coating	Covered with an acid-etched antireflective coating on all sides
Connected to aluminium box	Para rubber between the box and locking aluminium frame with 12 RVS A2 hexagonal recessed head screws (3.0 Ø × 20 mm)
**Operating system**	
Plateau with camera	2 Inch mini dome camera Wide angle camera without protective jacket IQLE, Model IQ667 (IQLE, Harderwijk, The Netherlands) Angle 130° Lens: 1.6 mm DC 12 V
Digital video recorder (DVR)	IQLE Recorder with 4 channels (Oosterberg B.V., Ede, The Netherlands) ½ D1 1TB hard disc IQ89000
Transceiver	225MPAS 1500MACT
Adapter	230 V–12 V

**Table 2:** Validation of the claw lesion scores obtained from the MCSD with soiled claws compared with the claw lesion scores obtained from the Feet First^©^ chute (“sow chute”, Zinpro Corporation) with cleaned claws as the gold standard. Values are least square means (LSmeans) (mm).

Hind Claw	Claw Lesion Type	Claw Digit	tVAS Score Sow Chute Cleaned	tVAS Score MCSD Soiled	Estimated Difference	CI
Left	Heel horn erosion	Lateral	87.67	86.23	1.43	[−3.09, 5.96]
		Medial	46.53 ^a^	60.94 ^b^	−14.41	[−18.89, −9.92]
	Separation along the heel-sole junction	Lateral	63.11	67.14	−4.03	[−11.01, 2.95]
		Medial	49.90 ^a^	58.93 ^b^	−9.03	[−15.61, −2.45]
	Separation along the white line	Lateral	72.28 ^b^	63.40 ^a^	8.89	[2.72, 15.05]
		Medial	42.41 ^a^	47.70 ^b^	−5.29	[−9.99, −0.59]
Right	Heel horn erosion	Lateral	88.33	87.30	1.03	[−4.43, 6.50]
		Medial	47.63 ^a^	59.48 ^b^	−11.85	[−16.72, −6.97]
	Separation along the heel-sole junction	Lateral	66.35	65.06	1.29	[−4.32, 6.90]
		Medial	50.36	53.52	−3.16	[−8.77, 2.44]
	Separation along the white line	Lateral	71.98 ^b^	65.64 ^a^	6.34	[0.94, 11.73]
		Medial	46.41	48.79	−2.38	[−8.01, 3.24]
Mean lateral claw digit			74.95	72.36	2.59	[−0.41, 5.60]
Mean medial claw digit			47.21 ^a^	55.44 ^b^	−8.23	[−11.12, −5.34]
Overall mean			61.08	63.53	−2.45	[−5.06, 0.16]

tVAS = tagged visual analogue scale for claw lesion scoring in sows as described in [9,10,31] and adapted and modified from the scorings methods of Wageningen University and FeetFirst (Zinpro Corporation, Eden Prairie, MN, USA). To score the claw area for claw lesions, a vertical bar was drawn on the line and the distance from 0 mm determined (0 mm is most healthy and 160 mm is most severe). MCSD = Mobile Claw Scoring Device. CI = confidence interval. FeetFirst^©^ chute, “sow chute”, Zinpro Corporation. Level of significance = 0.05 using a linear mixed model. ^a,b^ LSmean values within a row lacking common superscript letters differ significantly; *p* < 0.05.
